# Adherence to Cancer Prevention Guidelines among Older White and Black Adults in the Health ABC Study

**DOI:** 10.3390/nu11051008

**Published:** 2019-05-03

**Authors:** Audrey Y. Jung, Iva Miljkovic, Susan Rubin, Stephen B. Kritchevsky, Heidi D. Klepin, Anne B. Newman, Jane Cauley, Hilsa Ayonayon, Tamara B. Harris, Rachel A. Murphy

**Affiliations:** 1 School of Population and Public Health, University of British Columbia, Vancouver, BC V6T 1Z3, Canada; audrey.y.jung@gmail.com; 2 Center for Aging and Population Health, Department of Epidemiology, University of Pittsburgh, Pittsburgh, PA 15261, USA; MiljkovicI@edc.pitt.edu (I.M.); NewmanA@edc.pitt.edu (A.B.N.); JCauley@edc.pitt.edu (J.C.); 3 Department of Epidemiology and Biostatistics, University of California at San Francisco, San Francisco, CA 94158, USA; SRubin@psg.ucsf.edu (S.R.); HAyonayon@psg.ucsf.edu (H.A.); 4 Sticht Center for Healthy Aging and Alzheimer’s Prevention, Wake Forest School of Medicine, Winston-Salem, NC 27157, USA; skritche@wakehealth.edu; 5 Comprehensive Cancer Center, Wake Forest School of Medicine, Winston-Salem, NC 27157, USA; hklepin@wakehealth.edu; 6 Laboratory of Epidemiology and Population Sciences, National Institute on Aging, Bethesda, MD 20814, USA; harris99@nia.nih.gov

**Keywords:** lifestyle, nutrition, prevention, aging

## Abstract

One-third of cancers can be prevented through healthy lifestyles. This study investigates the prevalence of and factors associated with engagement in cancer prevention guidelines in a population-based cohort of 2124 older white and black men and women. We used Health ABC data to construct a score from 0 (lowest adherence) to 7 (greatest adherence) based on the sum of seven recommendations for cancer prevention from the World Cancer Research Fund/American Institute for Cancer Research; body fatness (maintenance of healthy body weight), physical activity (at least moderately physically active), diet (fruit, vegetables, fiber, and red and processed meat), and alcohol. Mean (SD) scores in men and women were 3.24 (1.09) and 3.17 (1.10). Lower scores were associated with younger age (women only), black race, current smoking, and prevalent cardiovascular disease. Less than 1% of men and women adhered to all recommendations. Of the individual guidelines, adherence was lowest for fiber (9% of men; 6% of women) followed by physical activity (26% of men; 18% of women), and body weight (21% of men; 26% of women). These results suggest a critical public health need, especially given the growing older population. Black older adults, smokers, and those with prevalent disease may be at higher risk and thus warrant additional focus.

## 1. Introduction

Cancer is a global problem and the burden of cancer is growing. In 2012, there were 14.1 million new cancer cases [1], which is projected to reach 22.2 million by 2030 [2]. The increase in cancer incidence is a reflection of population growth, the aging population and change in the prevalence and distribution of risk factors. The growing prevalence of obesity, a risk factor for numerous cancers, will have a major impact on cancer incidence in the coming decades [3]. Cancer prevention has thus been identified as a critical component of cancer control to mitigate the burden of cancer [4]. 

Lifestyle is a key component of cancer prevention strategies: one-third of cancers can be prevented by maintaining a healthy body weight, eating a healthy diet, and being physically active [5,6]. The World Cancer Research Fund (WCRF) and the American Institute for Cancer Research (AICR) provide evidence-based guidelines that focus on lifestyle recommendations to achieve and maintain a healthy body weight throughout life, adopt a physically active lifestyle, consume a healthy diet with an emphasis on plant foods, and limit alcohol consumption [7]. The recommendations include population goals and personal recommendations that have been used to inform policies and programs to reduce the incidence of cancer.

Despite the importance of lifestyle factors in cancer prevention, engagement in healthy lifestyle behaviors is low. A number of studies have examined the extent of adherence to preventive guidelines for cancer [8,9,10,11,12,13,14,15,16] in population-based cohorts of adults from different countries and consistently report low adherence to guidelines. However, there is limited information in older adults, despite more than half of all cancers occurring in people aged 65 and older [17]. 

The limited information may stem from the prevalent bias that older populations all suffer from chronic disease, and thus focus is directed towards disease management and not prevention of diseases [18] or that older adults are often excluded from randomized controlled trials of behavior change [19]. The time course of development for many cancers often exceeds a decade, suggesting that avoidance of risk accumulation must occur far before the onset of cancer [20]. Nevertheless, lifestyle behaviors are generally consistent over time and a greater understanding of cancer prevention is critically needed in older adults given the growing older population, and life expectancy that exceeds 10 years for many adults over age 65. Data from the Women’s Health Initiative suggests that this age range may be an etiologically relevant time period for cancer prevention. Women in the study who were aged 50–79 and had intentional weight loss, which tended to bring body weight in line with recommendations for a healthy body weight, had a lower risk of endometrial cancer [21]. Furthermore, a meta-analysis of seven cohorts, ranging in mean age from 60–70, showed that greater adherence to cancer prevention recommendations was associated with reduced cancer risk [16], thus suggesting older populations are not past a critical window for cancer prevention. 

The aim of this study was to provide observational data on the prevalence of engagement in guidelines for primary cancer prevention in a population-based cohort of older white and black men and women. Factors associated with adherence were also explored to provide insight into populations that may benefit from targeted cancer prevention strategies.

## 2. Research Design and Methods

### 2.1. Study Population 

Data for this study are from the Health, Aging, and Body Composition (Health ABC) Study, a prospective longitudinal cohort of 3075 community-dwelling participants aged 70–79 established in 1997–1998 [22]. Participants were recruited from a random sample of white Medicare-eligible residents and all black Medicare-eligible residents in the Pittsburgh, PA, and Memphis, TN, areas. Eligibility criteria included no difficulty walking one-quarter mile, climbing 10 steps without resting, or performing mobility-related activities of daily living, free of life-threatening illness, and no enrollment in lifestyle intervention trials. All participants provided written informed consent. Protocols were approved by the institutional review board at each study site.

Body weight and physical activity were assessed at multiple time points throughout the Health ABC Study. However, the food frequency questionnaire (FFQ) was only administered at the 12-month follow-up clinic visit (year 2). As dietary intake is a key component of cancer prevention guidelines, year 2 was selected as the analytical baseline, which also coincides with body weight and physical activity assessments. Participants with cancer diagnosed prior to the analytical baseline (*n* = 649) were excluded due to the potential influence of a cancer diagnosis on health behaviors and our study focus on adherence to recommendations for primary cancer prevention. Participants who developed nonmelanoma skin cancer prior to the analytical baseline were included as this form of cancer tends to behave very differently from other cancers (rarely metastasizes, high cure rate) and is treated differently. Participants who did not have any data on FFQ (*n* = 207) or BMI were also excluded (*n* = 95). Therefore, a total of 2124 persons were included.

### 2.2. Dietary Assessment

To estimate usual dietary intake, participants completed a 108-item interviewer-administered modified version of the Block FFQ (Block Dietary Data Systems, Berkeley, CA, USA) based on usual eating habits over the prior 12 months [23]. The FFQ food list was developed specifically for the Health ABC Study on the basis of 24-hour recall data from the National Health and Nutrition Examination Survey III for older black and white adults living in the Northeast or Southern US. Trained interviewers used wood blocks, food models, standard kitchen measures, and flash cards to help participants estimate predefined portion size categories for each food. Interviews were periodically monitored throughout the study to ensure the quality and consistency of the data collection procedures. Intakes of select food groups were calculated by Block Dietary Data Systems. Fiber was calculated as the sum of fiber from beans, grains and fruits and vegetables and questions included specifying white versus whole wheat breads and fiber or bran cereals. Red and processed meat included bacon, sausage, hamburgers, meat load, beef, pork, hot dogs, mixed meat dishes, and lunch meats. Alcohol consumption was estimated as the number of drinks per day from beer, wine and liquor. One standard drink was defined as beverages containing 0.6 oz or 14 g of pure alcohol e.g., 12-oz of beer (280 g), 5-oz of wine (117 g), or a 1.5-oz shot of liquor (35 g). A small amount of missing data from the FFQ’s (*n* = 11, 0.5% of participants for fruit and vegetables to *n* = 40, 1.8% for red meat) was imputed with the median intake of participants of the same gender, race and study site. 

### 2.3. Body Weight

Weight was measured at the analytical baseline using a calibrated scale. Height was measured using a stadiometer. Body mass index (BMI) was calculated in kg/m^2^. At the year 1 visit participants were asked to recall their usual weight at ages 25 and 50 years [24]. Studies in Health ABC suggest that these recalled weights are valid as they have been associated with weight-related outcomes such as mobility disability [24] and mortality [25] in comparable magnitude and direction as previous studies. Recalled height at age 25 years and measured height at Health ABC baseline were also highly correlated (*r* = 0.93, mean difference = 3 cm) as were self-reported weight and measured weight at the year 1 visit (*r* = 0.98).

### 2.4. Physical Activity

Physical activity was assessed using a self-reported questionnaire completed at the analytical baseline that was developed specifically for the Health ABC Study. The questionnaire was modeled after the validated Minnesota Leisure Time physical activity questionnaire [26]. Participants reported the type, intensity and amount of physical activity in the prior 7 days. The amount of moderate and vigorous physical activity was summed and considers physical activity and exercise in the context of this older population. For example, walking for exercise, other types of walking, and heavy chores that may be considered as light or lifestyle activity in a younger population are totaled with high intensity exercise (e.g., bicycling, swimming, jogging, or rowing) in the determination of moderate and vigorous physical activity. 

### 2.5. Adherence Score

Data collected in Health ABC pre-date the publication of the most recent WCRF/AICR guidelines (2018). However, as the recommendations build upon prior Expert Reports (2007 and 1997) without material changes to recommendations, the 2018 guidelines were used to facilitate comparison with future studies. Only recommendations that were quantifiable using data collected in Health ABC were included. For example, recommendations to consume fast food sparingly, consume energy-dense foods sparingly; limit refined starchy foods; avoid salt-preserved, salted, and salty foods; and avoid moldy cereals and pulses were not quantifiable with existing dietary data. In total, seven recommendations pertaining to maintenance of a body fatness, physical activity, plant foods, animal foods, and alcohol consumption were considered: maintenance of body weight within the normal range from age 21, avoid weight gain throughout adulthood, be at least moderately physically active, consume a diet with at least 30 g per day of fiber, include at least five portions/servings of nonstarchy vegetables and fruit every day, and limit consumption of red and processed meat. A composite score for adherence was generated in a manner similar to prior studies [13,27]. One point was assigned when the recommendation was met and zero points when it was not met. Criteria provided in the recommendations were used as cut points when provided (e.g., at least 5 servings of a variety of nonstarchy vegetables and fruit, <500 g/week of animal foods, and at least 30 g of fiber per day. Otherwise cut points previously defined in the literature were used. Details on the recommendations included in the study and the application of WCRF/AICR guidelines to the Health ABC population are shown in Table 1. The overall score ranged from 0 to 7 with high scores indicating greater adherence.

### 2.6. Statistical Analysis

Descriptive statistics are reported as means and standard deviations (SD) for continuous variables and as frequency and percentage for categorical variables. Linear regression models were used to determine adjusted regression coefficients and 95% confidence intervals (CIs) between participants’ composite score and potential associated factors known to be related to dietary intake and/or cancer incidence; age, race, education, smoking status, income, marital status, cardiovascular disease (coronary heart disease and stroke determined from self-report, hospitalization, and medications), and diabetes (determined from self-report, medications and clinical assessments). Logistic regression models were used to determine odds ratios (ORs) and 95% CIs between adherence to each of the component scores separately and potential associated factors as in the linear regression models. Regression models were adjusted for age, race, and study site, except the major independent variable. All analyses were stratified by sex due to significant interactions between sex and component measures; physical activity, body weight, fruits and vegetables, fiber and red and processed meat. *p*-values were two-tailed (α = 0.05). Analyses were performed with STATA software (version 14.1, StataCorp, College Station, TX, USA). In this cohort, the number of cancers that occurred after the analytical baseline (ascertained through ongoing cohort follow-up) is modest (295 in men and 222 in women), and were predominately prostate (*n* = 100), and lung (*n* = 99) cancers for which there is inconsistent evidence for lifestyle factors related to diet, weight and physical activity [28,29,30]. The next most common cancers were colon (*n* = 64) and breast (*n* = 57), which have more convincing relationships with lifestyle factors but the sample size is small. Thus, the association between adherence score and cancer incidence was not explored.

## 3. Results

Characteristics of the population are shown in Table 2. The mean age was 74.5 years (2.86 SD), 48% were men and 52% were women, and 34% of men and 44% of women were black. Most participants (48% of men and 40% of women) had at least a postsecondary education. Sixty percent of men and 34% of women were former smokers. Household income in men was predominately within $25,000 to $50,000 and $10,000 to $25,000 in women. Most men were married (75%) whereas most women (46%) were widowed. Over half of men and 44% of women reported prevalent cardiovascular disease. Prevalent diabetes was reported by 25% of men and 18% of women. 

The proportion of participants who adhered to individual cancer prevention recommendations are shown in Table 1. Adherence to recommendations for red and processed meat was highest (97% of men and 99% of women), followed by alcohol consumption (91% of men and 94% of women). Adherence was lowest for fiber (9% of men and 6% of women) followed by body weight for men (21%) and physical activity for women (18%). Nearly half of men and women met recommendations for fruit and vegetable consumption. Additional details on the dietary intake of foods/nutrients in the recommendations are provided in Supplementary Table S1.

The mean composite score, compiled from the individual recommendations showed low overall adherence (men: mean 3.24, SD 1.10; women: mean 3.17, SD 1.10). Frequencies of the composite score are shown in Table 3. All men and women met at least one recommendation. However, less than 1% of men and women met all seven recommendations.

The association between the composite cancer prevention score and characteristics of participants are presented in Table 4. Black men and black women’s adherence to prevention recommendations were 0.28 (SE = 0.07) and 0.43 (SE = 0.07) points lower than white men and women. In women, older age at analytical baseline was associated with a higher score (β = 0.03, SE=0.01) as was having a postsecondary education (β = 0.25, SE = 0.09). Women in Pittsburgh had lower scores than women in Memphis (β = −0.21, SE = 0.06) as did women with household income <$10,000 (β = −0.28, SE = 0.14). Current smokers had a composite prevention score 0.19 (men, SE 0.08) and 0.28 (women, SE 0.07) points lower than never smokers. Men and women with cardiovascular disease had a composite score that was 0.18 (SE 0.07) and 0.19 (SE 0.07) lower than those without cardiovascular disease. 

Odds ratios (OR) for each recommendation and associated factors are shown in Table 5. Associations for red and processed meat and alcohol consumption were not considered due to the low prevalence of not meeting guidelines. The odds of adherence to the body weight recommendation was 41% lower in men with diabetes, 72% lower among black women, and 60% lower in women with diabetes. Conversely, the odds of adherence to the body weight recommendation was 55% higher among women with a postsecondary education, as well as more than 2-fold higher among those with income less than $50,000. The odds of adherence to the weight maintenance recommendation was 31%, and 25% lower in men with diabetes and cardiovascular disease. Women from the Pittsburgh site, black women, current smokers and women with diabetes had 36%, 53%, 31%, and 48% lower odds of adherence to the weight maintenance recommendation, respectively. The odds of adherence to weight maintenance was more than 2-fold higher in men and women who were former smokers. The odds of meeting recommendations for physical activity were 31% and 54% lower among black men and women, 49% lower among men who were former smokers, and 41% and 37% lower among men and women with prevalent cardiovascular disease. Men from the Pittsburgh site, older women, and women with postsecondary education had greater odds of adherence to the recommendation for fruit and vegetables. Black men, women who were former smokers, and women with cardiovascular disease had 45%, 44%, and 31% lower odds of adherence to the recommendation for fruit and vegetables, respectively. Adherence to the recommendation for fiber was 41% lower among women from the Pittsburgh site, 38% lower among men who were current smoker, and more than 3-fold higher among never married men.

## 4. Discussion and Implications

This study adds to the body of evidence on adherence to cancer prevention guidelines by contributing information on populations for which there is limited evidence; older adults and black participants. Our results show that less than 1% of men and women adhered to all prevention guidelines assessed in our study, and overall adherence to prevention guidelines is particularly low among black participants and men. Conversely, guidelines for alcohol and red and processed meat consumption were followed by the majority of participants. This along with the variation in adherence to each cancer prevention guidelines suggests some guidelines may be easier to achieve than others. 

In this cohort, adherence to fiber (9% and 6%), was the lowest of the recommendations assessed. This is consistent with data from the 2009–2010 National Health and Nutrition Examination Survey that illustrated low fiber and whole grain intake among the general US population [31], as well as fiber being identified as a nutrient of public health concern by the 2015 Dietary Guidelines for Americans due to low intake [32]. The WCRF/AICR grades the evidence linking wholegrains and foods containing dietary fiber to colorectal cancers as ‘strong’ [7], suggesting the need for continual public health efforts to increase consumption, particularly given that colorectal cancer is the fourth most common cancer diagnosis in the United States and the second leading cause of cancer related deaths [17]. 

Adherence to the physical activity recommendation was also low in this cohort (26% and 18%). This may reflect the older age of the cohort as physical activity levels tend to decline with age [33]. It is also noteworthy that adherence was low despite a population that was well-functioning and physical activity was self-reported, which is prone to response bias [34]. Indeed, among adults aged 70 or older, only 6% to 10% meet recommendations for physical activity of ≥150 minutes of MVPA/wk using accelerometry measured physical activity [35], which is lower than the cut point of ≥ 210 minutes of MVPA/wk in our study. It is thus likely that adherence to physical activity guidelines in our population as well as the more general older population is even less than what we report. 

Approximately one in four participants adhered to the guidelines for weight maintenance within the normal range throughout life, and upwards of 70% of men and 80% of women gained more than 5% of their body weight as they aged. This reflects the high prevalence of overweight and obesity in the United States; over 70% among adults age 60 and older [36] and the predominant experience of weight gain with aging [37]. Body fatness is one of the strongest and most universal risk factor for cancer (as well as other chronic disease). Body fatness has been linked to twelve different types of cancer for which the evidence is rated as ‘probable increased risk’ for three cancers to ‘convincing increased risk’ for eight cancers which is the highest grade of evidence from the WCRF/AICR [7]. Obesity has been the focus of many public health efforts in the United States [38,39,40], however obesity rates remain high. More work is needed to develop effective strategies with a consideration of interventions that address preventable health behaviors among older populations.

Several factors—black race, former and current smoking, lower education and income, cardiovascular disease, and diabetes—were associated with lower likelihood of meeting individual prevention guidelines and/or having a lower composite score. Although former smoking was also associated with greater odds of weight maintenance, likely reflecting well-established relationships between lower weight and smoking. Our findings are consistent with well-known racial/ethnic and socioeconomic health disparities in health risk behaviors in the United States [41,42]. Cardiovascular disease and diabetes share many of the same modifiable risk factors as cancer including body fatness, physical activity, and some aspects of diet [43,44,45,46]. Thus, it is not surprising that participants with prevalent cardiovascular disease and diabetes were less likely to adhere to guidelines. Rather, this provides insight into specific populations who may be particularly at risk for cancer, and thus would benefit from targeted cancer prevention strategies. 

Direct comparison with previous studies is limited by the challenges in assessing adherence to the WCRF/AICR recommendations. Clear cut points do not exist for all recommendations such as sedentary time, wholegrains, pulses, and non-starchy vegetables. Recommendations that include ‘limit’ or ‘consume sparingly’ are difficult to operationalize and compare across studies and the number of individual recommendations assessed in a given study is variable. That said, the overall finding of low engagement in cancer preventive behavior is consistent with previous studies across a variety of populations from different countries. Among men and women aged 50–71 [10], 12% of men and 11% of women were in the lowest category of adherence. A study of women aged 50–79 [15] reported less than 1% of participants met all guidelines and similar to our findings reported lower adherence scores among women with less education, former or current smokers and black women. Jankovic et al. [16] reported adherence to recommendations for fiber, fruits, and vegetables and alcohol consumption that were in the same range as our results across the six older cohorts studied. Compared to studies of adherence in younger populations [9,13,27,47], adherence to the body weight recommendation was consistently lower in our population, adherence to physical activity and fiber and alcohol recommendations were within the ranges previously reported and adherence to the recommendation for fruits and vegetables, red and processed meat were generally higher. Additional studies are needed to confirm whether older adults truly have a higher/lower adherence to specific recommendations than younger populations or whether differences reflect heterogeneity in study methods and cutpoints. Understanding meaningful differences in populations may help to focus prevention efforts. 

A strength of our study is the recalled height and weight at ages 25 and 50. This enabled the assessment of maintenance of healthy body weight over time, which few prior studies have done. Another strength of our study is the population of older, black, and white adults, demographics that have been seldom captured in prior studies. However, since this study was confined to a specific age group, and data were collected in 1998–1999, the results may not be generalizable to contemporary older adults, particularly given the increase in body weight over the past two decades [48]. Participants were also recruited to be initially well functioning and thus selection bias may mean that the results do not reflect the larger population of 71–80 year olds regardless of the era of assessment. Diet, physical activity and alcohol were only assessed at a single time point, which may not capture variation. There are also limitations to our approach to operationalize the guidelines. We relied on previously published cut points for recommendations without well-defined criteria. As well, the WCRF/AIRC does not provide guidance on relative importance of recommendations, and the complexity of such an undertaking would be substantial. As a result, each individual component received an equal weight within the composite score even though the attributable risk of each factor may vary or only be associated with certain cancer types [7]. Additional work to facilitate comparable surveillance data on cancer preventive behaviors is needed.

In conclusion, our results suggest that few older adults adhere to guidelines for cancer prevention, thus illustrating the critical need for cancer prevention efforts in the rapidly aging population. Adherence was related to socioeconomic factors, race, and prevalent disease. Some guidelines such as fiber, physical activity and weight may be particularly difficult to meet. These findings may be helpful for informing cancer prevention strategies including identifying priority populations and behaviors to target. 

## Figures and Tables

**Table 1:** WCRF/AICR personal recommendations for cancer prevention and translation to adherence score in the Health ABC Study.

Personal Recommendations	Operationalization	Score	Men *n* (%)	Women *n* (%)
Body fatness				
Maintain body weight within the normal range from age 21 *	All other combinations	0	802 (79.2)	822 (73.9)
	BMI 18.5 to 24.9 kg/m^2^ at 25y, 50y and 71-80y	1	210 (20.8)	290 (26.1)
Avoid weight gain throughout adulthood *	≥5% weight gain	0	689 (68.1)	879 (79.1)
	<5% weight gain	1	323 (31.9)	233 (21.0)
Physical activity *				
Be at least moderately physically active and follow or exceed national guidelines	<210 minutes of moderate to vigorous physical activity/wk	0	747 (73.8)	907 (81.6)
	≥210 minutes of moderate to vigorous physical activity/wk	1	265 (26.2)	205 (18.4)
Plant foods				
Eat at least five portions/servings of a variety of nonstarchy vegetables and fruit every day	<5 servings of fruits and vegetables/d over last 12 months	0	524 (51.8)	522 (46.9)
	≥5 servings of fruits and vegetables/d over last 12 months	1	488 (48.2)	590 (53.1)
Consume a diet that provides at least 30 g per day of fiber from food sources *	Dietary fiber intake <30 g/d	0	917 (90.6)	1045 (94.0)
	Dietary fiber intake ≥30 g/d	1	95 (9.39)	67 (6.03)
Animal foods *				
Limit consumption of red meat to no more than 500 g a week, eat little if any processed meat	≥500 g red meat/wk and/or processed meat ≥50 g/d	0	30 (2.96)	15 (1.35)
	<500 g red meat/wk and <50 g/d processed meat/s	1	982 (97.0)	1097 (98.7)
Alcoholic drinks *				
If consumed, do not exceed national guidelines	>2 drinks/d for men and >1 drink/d for women	0	93 (9.19)	65 (5.8)
	≤2 drinks/d for men and ≤1 drink/d for women	1	919 (90.8)	1047 (94.2)

* Difference between men and women *p* < 0.05 from chi-square tests.

**Table 2:** Characteristics of participants in the Health ABC Study by sex.

	Men (*n* = 1012)	Women (*n* = 1112)
Age, mean (SD)	74.7 (2.85)	74.4 (2.87)
Black, *n* (%)	342 (33.8)	480 (43.2)
Pittsburgh site, *n* (%)	506 (50)	577 (51.9)
Education, *n* (%)		
<High school	260 (25.7)	235 (21.2)
High school	264 (26.1)	428 (38.6)
Postsecondary	486 (48.1)	447 (40.3)
Smoking status, *n* (%)		
Never	300 (29.7)	641 (57.7)
Current	102 (10.1)	97 (8.73)
Former	608 (60.2)	373 (33.6)
Household income, *n* (%)		
<$10,000	63 (6.90)	163 (17.1)
$10,000 to $25,000	313 (34.3)	399 (41.9)
>$25,000 to <$50,000	337 (36.9)	277 (29.1)
≥$50,000	200 (21.9)	114 (12.0)
Marital status, *n* (%)		
Married	712 (74.6)	397 (38.9)
Widowed	129 (13.5)	467 (45.7)
Divorced/separated	71 (7.4)	105 (10.3)
Never married	42 (4.40)	53 (5.19)
Cardiovascular disease, *n* (%)	570 (56.8)	483 (44.2)
Diabetes, *n* (%)	254 (25.1)	204 (18.4)

**Table 3:** Prevalence of adherence to cancer prevention guidelines by sex in the Health ABC Study, n (%).

	0	1	2	3	4	5	6	7
Men	0	26 (2.57)	246 (24.3)	356 (35.2)	251 (24.8)	108 (10.7)	23 (2.27)	2 (0.20)
Women	0	19 (1.71)	309 (27.8)	421 (37.9)	227 (20.4)	100 (8.99)	31 (2.79)	5 (0.45)

**Table 4:** Association between the composite cancer prevention score and characteristics in the Health ABC Study by sex.

	Men		Women	
	β (SE)	*p*-Value	β (SE)	*p*-Value
^a^ Age	0.01 (0.01)	0.24	0.03 (0.01)	0.004
Memphis site	Reference		Reference	
Pittsburgh site	−0.05 (0.07)	0.47	−0.21 (0.06)	0.001
White race	Reference		Reference	
Black race	−0.28 (0.07)	<0.001	−0.43 (0.07)	<0.001
Education				
<High school	Reference		Reference	
High school	−0.12 (0.10)	0.24	0.07 (0.09)	0.44
Postsecondary	0.12 (0.09)	0.20	0.25 (0.09)	0.006
Smoking status				
Never	Reference		Reference	
Former	−0.13 (0.13)	0.30	0.11 (0.12)	0.35
Current	−0.19 (0.08)	0.02	−0.28 (0.07)	<0.001
Household income				
<$10,000	0.02 (0.17)	0.90	−0.28 (0.14)	0.04
$10,000 to $25,000	−0.01 (0.10)	0.91	−0.18 (0.12)	0.12
>$25,000 to <$50,000	0.00 (0.10)	0.97	−0.18 (0.12)	0.14
≥$50,000	Reference		Reference	
Marital status				
Married	Reference		Reference	
Widowed	−0.02 (0.11)	0.86	−0.04 (0.08)	0.62
Divorced/separated	−0.17 (0.14)	0.22	−0.10 (0.12)	0.43
Never married	0.07 (0.17)	0.67	−0.06 (0.16)	0.71
Cardiovascular disease	−0.18 (0.07)	0.01	−0.19 (0.07)	0.005
Diabetes	−0.15 (0.08)	0.07	−0.15 (0.08)	0.08

^a^ Coefficient represents change in one year, models adjusted for age, race, and study site. Greater composite scores represent greater adherence to recommendations.

**Table 5:** Associations between adherence to individual cancer prevention guidelines and characteristics in the Health ABC Study by sex.

	Men		Women	
	OR (95% CI)	*p*-Value	OR (95% CI)	*p*-Value
*Body weight*				
Age	1.04 (0.98–1.09)	0.19	1.04 (0.99–1.09)	0.10
Memphis site	Reference		Reference	
Pittsburgh site	0.95 (0.70–1.28)	0.72	0.89 (0.67–1.17)	0.39
White race	Reference		Reference	
Black race	0.75 (0.53–1.04)	0.08	0.28 (0.21–0.38)	9.7 × 10^−16^
Education				
<High school	Reference		Reference	
High school	1.19 (0.75–1.90	0.46	1.05 (0.68–1.62)	0.83
Postsecondary	1.48 (0.97–2.27)	0.07	1.55 (1.02–2.38)	0.04
Smoking status				
Never	Reference		Reference	
Former	1.56 (0.90–2.69)	0.11	1.59 (0.98–2.59)	0.06
Current	0.97 (0.69–1.37)	0.86	0.85 (0.63–1.16)	0.31
Household income				
<$10,000	1.01 (0.49–2.09)	0.99	2.03 (1.18–3.50)	0.01
$10,000 to $25,000	1.16 (0.55–2.44)	0.70	2.07 (1.16–3.67)	0.01
>$25,000 to <$50,000	1.11 (0.51–2.43)	0.79	2.36 (1.24–4.52)	0.01
≥$50,000	Reference		Reference	
Marital status				
Married	Reference		Reference	
Widowed	0.84 (0.52–1.36)	0.48	0.94 (0.68–1.31)	0.73
Divorced/separated	0.51 (0.24–1.09)	0.08	0.63 (0.35–1.15)	0.13
Never married	0.84 (0.38–1.86)	0.67	0.98 (0.51–1.86)	0.94
Cardiovascular disease	0.80 (0.59–1.10)	0.17	0.86 (0.64–1.14)	0.29
Diabetes	0.59 (0.40–0.87)	0.01	0.40 (0.26–0.63)	<0.001
*Weight maintenance*				
Age	1.03 (0.99–1.08)	0.18	1.08 (1.02–1.13)	0.004
Memphis site	Reference		Reference	
Pittsburgh site	0.95 (0.73–1.23)	0.68	0.64 (0.48–0.86)	0.003
White race	Reference		Reference	
Black race	1.11 (0.84–1.47)	0.45	0.47 (0.35–0.65)	<0.001
Education				
<High school	Reference		Reference	
High school	0.70 (0.47–1.03)	0.07	0.92 (0.59–1.45)	0.73
Postsecondary	0.92 (0.65–1.30)	0.63	1.40 (0.90–2.16)	0.13
Smoking status				
Never	Reference		Reference	
Former	2.22 (1.39–3.55)	0.001	3.24 (2.03–5.17)	<0.001
Current	0.80 (0.60–1.08)	0.15	0.69 (0.49–0.98)	0.04
Household income				
<$10,000	1.04 (0.58–1.87)	0.89	0.96 (0.59–1.57)	0.87
$10,000 to $25,000	0.84 (0.46–1.55)	0.58	0.92 (0.54–1.57)	0.75
>$25,000 to <$50,000	0.83 (0.43–1.57)	0.56	0.95 (0.50–1.80)	0.88
≥$50,000	Reference		Reference	
Marital status				
Married	Reference		Reference	
Widowed	1.15 (0.77–1.72)	0.48	0.83 (0.59–1.18)	0.30
Divorced/separated	0.72 (0.41–1.27)	0.26	0.77 (0.42–1.40)	0.39
Never married	1.07 (0.55–2.08)	0.83	1.22 (0.63–2.36)	0.55
Cardiovascular disease	0.75 (0.57–0.95)	0.04	0.91 (0.67–1.23)	0.52
Diabetes	0.69 (0.50–0.95)	0.02	0.52 (0.33–0.82)	0.005
*Physical Activity*				
Age	0.98 (0.94–1.03)	0.51	0.95 (0.90–1.00)	0.07
Memphis site	Reference		Reference	
Pittsburgh site	0.78 (0.59–1.03)	0.08	0.63 (0.46–0.85)	0.003
White race	Reference		Reference	
Black race	0.69 (0.51–0.94)	0.02	0.46 (0.33–0.64)	<0.001
Education				
<High school	Reference		Reference	
High school	0.96 (0.64–1.46)	0.86	1.17 (0.74–1.86)	0.50
Postsecondary	1.01 (0.70–1.48)	0.94	1.04 (0.65–1.65)	0.88
Smoking status				
Never	Reference		Reference	
Former	0.51 (0.28–0.92)	0.03	1.35 (0.80–2.29)	0.26
Current	0.93 (0.68–1.27)	0.65	0.74 (0.53–1.05)	0.10
Household income				
<$10,000	1.18 (0.60–2.33)	0.64	1.16 (0.69–1.97)	0.58
$10,000 to $25,000	1.38 (0.69–2.78)	0.37	1.11 (0.62–1.96)	0.73
>$25,000 to <$50,000	1.28 (0.62–2.67)	0.51	1.29 (0.67–2.51)	0.45
≥$50,000	Reference		Reference	
Marital status				
Married	Reference		Reference	
Widowed	0.72 (0.45–1.15)	0.17	1.04 (0.73–1.50)	0.82
Divorced/separated	1.15 (0.66–2.01)	0.63	0.82 (0.44–1.52)	0.52
Never married	0.62 (0.28–1.37)	0.24	1.00 (0.48–2.11)	0.99
Cardiovascular disease	0.59 (0.44–0.79)	<0.001	0.63 (0.45–0.87)	0.01
Diabetes	0.82 (0.59–1.15)	0.25	0.92 (0.61–1.41)	0.71
*Fruits and Vegetables*				
Age	1.01 (0.97–1.06)	0.56	1.05 (1.00–1.09)	0.04
Memphis site	Reference		Reference	
Pittsburgh site	1.30 (1.01–1.66)	0.04	0.92 (0.73–1.17)	0.48
White race	Reference		Reference	
Black race	0.55 (0.42–0.72)	<0.001	0.87 (0.68–1.10)	0.25
Education				
<High school	Reference		Reference	
High school	0.75 (0.52–1.09)	0.13	1.17 (0.84–1.64)	0.36
Postsecondary	1.14 (0.82–1.59)	0.44	1.80 (1.28–2.54)	0.001
Smoking status				
Never	Reference		Reference	
Former	0.64 (0.40–1.04)	0.07	0.56 (0.36–0.87)	0.01
Current	0.85 (0.64–1.12)	0.24	0.82 (0.64–1.06)	0.13
Household income				
<$10,000	0.75 (0.43–1.32)	0.32	1.04 (0.72–1.51)	0.83
$10,000 to $25,000	0.74 (0.41–1.32)	0.31	1.29 (0.85–1.96)	0.23
>$25,000 to <$50,000	0.90 (0.49–1.67)	0.74	2.15 (1.26–3.66)	0.005
≥$50,000	Reference		Reference	
Marital status				
Married	Reference		Reference	
Widowed	0.88 (0.60–1.30)	0.52	0.85 (0.64–1.13)	0.25
Divorced/separated	0.79 (0.47–1.33)	0.38	1.10 (0.71–1.72)	0.67
Never married	1.26 (0.67–2.36)	0.48	0.76 (0.43–1.36)	0.36
Cardiovascular disease	0.89 (0.69–1.16)	0.40	0.69 (0.54–0.88)	0.003
Diabetes	1.06 (0.79–1.42)	0.69	1.22 (0.89–1.66)	0.22
*Fiber*				
Age	0.95 (0.88–1.02)	0.14	1.07 (0.99–1.17)	0.10
Memphis site	Reference		Reference	
Pittsburgh site	0.95 (0.62–1.45)	0.81	0.51 (0.30–0.85)	0.009
White race	Reference		Reference	
Black race	0.98 (0.63–1.53)	0.93	1.59 (0.96–2.62)	0.07
Education				
<High school	Reference		Reference	
High school	0.99 (0.51–1.93)	0.98	0.77 (0.38–1.55)	0.46
Postsecondary	1.52 (0.45–2.70)	0.16	1.20 (0.62–2.33)	0.59
Smoking status				
Never	Reference		Reference	
Former	0.57 (0.25–1.30)	0.18	0.57 (0.20–1.64)	0.30
Current	0.62 (0.39–0.98)	0.04	0.72 (0.41–1.27)	0.25
Household income				
<$10,000	1.04 (0.37–2.95)	0.94	0.64 (0.24–1.74)	0.38
$10,000 to $25,000	0.96 (0.50–1.82)	0.89	0.49 (0.21–1.18)	0.11
>$25,000 to <$50,000	1.23 (0.68–2.23)	0.50	0.55 (0.22–1.34)	0.19
≥$50,000	Reference		Reference	
Marital status				
Married	Reference		Reference	
Widowed	0.95 (0.47–1.91)	0.88	1.61 (0.89–2.90)	0.12
Divorced/separated	1.55 (0.72–3.33)	0.36	1.12 (0.43–2.95)	0.82
Never married	3.85 (1.84–8.05)	<0.001	0.77 (0.17–3.43)	0.73
Cardiovascular disease	0.83 (0.54–1.29)	0.41	1.26 (0.76–2.10)	0.37
Diabetes	1.22 (0.76–1.96)	0.41	1.68 (0.95–2.96)	0.07

OR represents the odds of meeting cancer prevention recommendations. Models adjusted for age, race, and study site.

## Supplementary Materials

The following are available online at https://www.mdpi.com/2072-6643/11/5/1008/s1, Table S1: Dietary intake (Mean, (SD)) in Health ABC of foods considered in the composite score of WCRF/AICR dietary recommendations.
