# Supplementary material for: Adherence to Cancer Prevention Guidelines among Older White and Black Adults in the Health ABC Study
Source: Nutrients. 2019 May 3;11(5):1008. doi: 10.3390/nu11051008 (PMC6566295; doi:10.3390/nu11051008)
Supplement: Supplementary file 1 [file nutrients-11-01008-s001.zip › Supplementary Table 1.docx]

**Supplementary Table 1.** Dietary intake (Mean, (SD)) in Health ABC

of foods considered in the composite score of WCRF/AICR dietary recommendations

|  | Men | Women |
| --- | --- | --- |
| Daily servings of fruit | 1.79 (1.12) | 3.00 (1.94) |
| Daily servings of vegetables | 2.99 (1.93) | 3.00 (1.94) |
| Daily total dietary fiber (g) | 18.6 (8.45) | 16.9 (7.41) |
| Daily dietary fiber from beans (g) | 2.70 (3.04) | 2.10 (2.72) |
| Daily dietary fiber from vegetables and fruit (g) | 6.88 (3.95) | 7.26 (3.98) |
| Daily dietary fiber from grains (g) | 9.36 (4.96) | 7.80 (4.06) |
| Weekly red meat intake (g) | 518 (464) | 347 (356) |
| Daily processed meat intake (g) | 14.4 (16.0) | 9.23 (12.2) |
